# The Influence of Apical and Basal Defoliation on the Canopy Structure and Biochemical Composition of *Vitis vinifera* cv. Shiraz Grapes and Wine

**DOI:** 10.3389/fchem.2017.00048

**Published:** 2017-07-07

**Authors:** Pangzhen Zhang, Xiwen Wu, Sonja Needs, Di Liu, Sigfredo Fuentes, Kate Howell

**Affiliations:** School of Agriculture and Food, University of MelbourneParkville, VIC, Australia

**Keywords:** canopy management, defoliation, canopy structure, image analysis, shiraz, wine, aroma profile

## Abstract

Defoliation is a commonly used viticultural technique to balance the ratio between grapevine vegetation and fruit. Defoliation is conducted around the fruit zone to reduce the leaf photosynthetic area, and to increase sunlight exposure of grape bunches. Apical leaf removal is not commonly practiced, and therefore its influence on canopy structure and resultant wine aroma is not well-studied. This study quantified the influences of apical and basal defoliation on canopy structure parameters using canopy cover photography and computer vision algorithms. The influence of canopy structure changes on the chemical compositions of grapes and wines was investigated over two vintages (2010–2011 and 2015–2016) in Yarra Valley, Australia. The Shiraz grapevines were subjected to five different treatments: no leaf removal (Ctrl); basal (TB) and apical (TD) leaf removal at veraison and intermediate ripeness, respectively. Basal leaf removal significantly reduced the leaf area index and foliage cover and increased canopy porosity, while apical leaf removal had limited influences on canopy parameters. However, the latter tended to result in lower alcohol level in the finished wine. Statistically significant increases in pH and decreases in TA was observed in shaded grapes, while no significant changes in the color profile and volatile compounds of the resultant wine were found. These results suggest that apical leaf removal is an effective method to reduce wine alcohol concentration with minimal influences on wine composition.

## Introduction

Climate change has already had many impacts on the global wine industry, some of them that could be considered beneficial or detrimental depending on the growing region (Jones and Webb, 2010; Mira De Orduna, 2010; Mozell and Thach, 2014). The phenological development of grapevines is altered mainly due to the changes in global temperature, carbon dioxide concentration [CO_2_], and solar radiation, leading to compression of phenological stages, accelerated grape maturation and earlier harvest dates (Mira De Orduna, 2010). One of the major consequences to grape chemistry is the elevated berry sugar level, which is mainly caused by excessive evaporative loss of water, rather than increased photosynthesis (Keller, 2010; Mozell and Thach, 2014). Elevated wine alcohol levels are reported in major existing wine regions of the world (Jones, 2007; Van Leeuwen and Darriet, 2016). Increasing grape sugar and consequently wine alcohol levels is deemed undesirable for the wine industry, which can be related to a range of problems, such as: stuck alcoholic fermentation, increased levels of acetic acid, higher energy requirements for cooling, generation of unwanted by-products, alteration of wine sensory profile, potential influences on human health and reducing competitiveness due to alcohol associated taxation (Erasmus et al., 2003; Coulter et al., 2008; Mira De Orduna, 2010; Ashenfelter and Storchmann, 2016). Thus, techniques controlling sugar concentration in grapes and wine alcohol levels with minimal influences on wine biochemical profile are favored by the wine industry in general.

Four major approaches may be used to reduce alcohol levels in wine: (i) microbiological interventions; (ii) genetic engineering of fermenting yeast; (iii) physical/enzymatic treatment of grapes and wine and (iv) agronomical management in the vineyard. The microbiological approach selects yeast strains with lower ethanol yield during the alcoholic fermentation. Milanovic et al. (2012) observed reduced ethanol production with the co-fermentation of immobilized *Strarmerella bombicola* and *Saccharomyces cerevisiae*. Genetic engineering techniques could control the genes expression of *S. cerevisiae*, and therefore convert sugar into other by-product and create new yeast strains (Ozturk and Anli, 2014).

Physical or enzymatic removal of ethanol can also effectively reduce alcohol level in wine. For example, membrane-based systems are now commonly used in new world wine countries, however, may have some adverse impacts on the aroma of wine (Diban et al., 2008). On the other hand, adding the enzyme glucose oxidase (GOX) during fermentation from the fungus *Aspergillus niger* catalyzes the conversion of glucose into gluconic acid and hydrogen peroxide, thus leading to lower alcohol in the resultant wine (Biyela et al., 2009; Ozturk and Anli, 2014), but this intervention is controlled in many winegrowing regions.

Grapevine canopy management has been commonly used to control sugar accumulation during grape maturation to achieve lower alcohol wines. The rate of sugar accumulation in berries is largely dependent on the ratio of leaf area to fruit weight (LA/FW), and lower sugar accumulation in grape berries can be achieved by reducing leaf area (Ozturk and Anli, 2014). The influences of leaf trimming on grape biochemical composition has been researched on many grapevine cultivars, while limited research has been conducted on *V. vinifera* cv. Shiraz, which is one of the major cultivars planted in Australia (Kozina et al., 2008; Lanari et al., 2013; Baiano et al., 2015; Feng et al., 2015; Osrečak et al., 2016). In addition, defoliation studies tend to focus on bunch zone leaf removal, which not only modifies the LA/FW ratio, but also increases the direct solar exposure and alter bunch zone microclimate (Spayd et al., 2002). The influence of apical leaf trimming on the canopy structure and biochemical composition of resultant grapes and wines has not been well-studied.

The objective of this research was to quantify the impacts of apical and basal leaf removal at different grapevine phenological stages on the canopy structure of grapevines. Furthermore, this research investigates the consequences of leaf removal on the biochemical composition of grape berries and wine in *V. vinifera* cv. Shiraz. The findings of this project provide vineyard managers an alternative canopy management method to manipulate grape sugar accumulation and to reduce wine alcohol levels with minimal influences on the wine volatile profile.

## Materials and methods

### Vineyard site

This study was conducted on a commercial Shiraz (*Vitis vinifera* cv.) vineyard located at Coldstream, Victoria, Australia (latitude −37.72, longitude 145.41, elevation 83 m) in two contrasting growing seasons (2010–2011 and 2015–2016) The weather data was obtained from the closest Bureau of Meteorology weather station (~1.5 km; BOM weather station No. 086383). The mean January temperature (MJT) and total rainfall from budburst (October) to harvest for the two studied growing seasons were 20.4°C; 706.8 mm and 20.7°C; 204 mm, respectively. Grapevines were trained using a vertical shooting positioning trellis system. All vine rows were orientated from north to south. A total of 25 panels of vines were established in each row with 4 vines in each panel. Vine spacing was 2.8 m between rows and 1.8 m between vines. Canopy management practices were restricted to the treatments from this trial. Other agronomical management practices were applied with normal commercial standards. No specific pest and disease pressure was observed during both experimental seasons.

### Experimental design and berry measurements

*Vitis vinifera* L. cv. Shiraz grapevines included in this study were subjected to five treatments: (i) no defoliation treatment (Ctrl); (ii) 5–7 basal leaves surrounding bunches removed from each shoot at Veraison (0–50%) (TB-v) or at mid ripeness (TB-m); (iii) seven fully expanded apical leaves removed from each shoot at Veraison (0–50%) (TD-v) or mid ripeness (TD-m) in the 2015–2016 growing season, and only Ctrl and TA-v treatment in the 2011–2012 growing season. Five replicates were conducted for each treatment with one panel of grapevines as one individual replicate in the 2015–2016 growing season, while 12 replicates were conducted in 2011–2012, following a completely random block design.

Grape samples (200 g per replicate) were collected in zip-lock plastic bags before treatment at baseline, then fortnightly since treatment until commercial harvest on 5th April 2011 and 2nd March 2016. For each sampling date, fruit was transferred to the laboratory of the winery for immediately analysis in the same day (2011–2012). Samples were stored at −20°C in the winery laboratory, and later on transferred the University of Melbourne in Styrofoam boxes on dry ice, then stored at −20°C until analysis (2015–2016). In the 2011–2012 growing season, grape samples from individual replicates were blended together and subjected to chemical analysis. Total soluble solid (TSS, °Brix), pH, titratable acidity (TA), total anthocyanins, and total phenolics were analyzed using a refractometer, pH meter, alkaline titration, and spectrophotometer, respectively, following the published protocol (Iland, 2004).

### Leaf area index (LAI) and canopy structure measurements

All the experimental replicates were subjected to canopy structure measurements at mid-ripeness after defoliation treatments in the 2015–2016 growing season following the canopy cover photography method (Fuentes et al., 2008, 2014). For this, an iPhone 5s (Apple lnc. Cupertino, CA, USA) was used to acquire digital images of grapevine canopy using the back camera. Upward-looking images were obtained from under the grapevine canopies at 0° zenith angle and around 20 cm above ground. Three images were obtained from each treated panel around the middle grapevine. Images were labeled and saved in fine JPEG format. The images collected were then analyzed using computer vision algorithms through a customized code written in Matlab. v2015b (Mathworks Inc., Matick, MA, USA; Fuentes et al., 2008, 2014).

### Microvinification

Additional 2 kg of grape bunches were collected from each replicate at harvest and cooled down to 4°C on dry ice to be transferred to the laboratory. Small scale fermentation was performed following the protocol established by the Irymple Research Centre of the Department of Economics, Development, Jobs, Transport, and Resources of the Victorian Government (DEDJTR) as described earlier (Kilmister et al., 2014). Briefly, harvested grapes were destemmed, crushed in sanitized 1.5–2 L containers, pH of the musts were adjusted to 3.4–3.5 with tartaric solution (10%), then diammonium phosphate (10%) at 1 ml/L of juice was added plus commercial yeast at 0.2 g/L (EC1118). Primary fermentation in microvinification were done at 18°C in a temperature controlled room for 7 days until Baume readings reached 1–2 for pressing using a ratchet style press. Wines were transferred into clean 500 ml glass bottles, with addition of potassium metabisulfite (10%) at 0.5 ml/L and copper sulfate (0.4%) at 1 ml/L, and placed in at 16°C controlled cool room for 14 days. After, wines were racked into new bottles filled with CO_2_, and placed in a 1–2°C cool room for stabilization. Wines were then bottled into clean 375 ml bottles and placed in 14°C cool room until analysis.

#### Preparation of samples and headspace solid-phase microextraction and gas chromatography mass spectrometry (HS-SPME-GC-MS) analysis of wine volatiles

Wine samples were prepared for wine volatile analysis based on the protocol proposed by Siebert et al. (2005) with some modifications. For this, 1 ml of wine samples were diluted with 9 ml of milli Q water into HS-SPME vial (Agilent Technologies, 20 ml) with addition of 2 g of sodium chloride and 200 μL of 4-octanol (Internal standard; 10 mg/L) and ethyl non-anoate (quality control; 10 ml/L). The vial and its contents were shaken at 220 rpm and heated to 35°C. A polydimethylsiloxane (PDMS, Agilent) 100 μm fiber was exposed to the headspace and agitated for 10 min.

An Agilent Technologies 6850 series II (GC; Agilent Technologies, Santa Clara, CA) equipped with an Agilent PAL 120 multipurpose auto-sampler and coupled with an Agilent 5,873 mass selective detector (MSD) was used for volatile assessments. The instruments were controlled using Agilent G1701EA MSC ChemStation software in conjunction with Agilent PAL Sampler Software Control B.01.04 for ChemStation. The GC was fitted with a J&W DB-wax column (30 m × 0.25 mm, 0.25 μm film df) with helium as carrier gas (ultrahigh purity, BOC Australia, North Ryde, NSW, Australia), and the flow rate was 2.0 ml/min in constant flow mode. The GC inlet was fitted with a borosilicate glass SPME inlet liner (Agilent, 6.3 mm o.d., 78.5 mm length) held at 220°C.

The SPME fiber was desorbed in the pulsed splitless mode and the splitter at 50:1, was opened after 30 s. The fiber was allowed to bake in the inlet for 10 min. The oven was started at 40°C, held for 4 min, then increased to 220°C at 5°C, and held for 20 min. The MS source, quadruple and transfer line was held at 230°C, 106°C, and 250°C, respectively. The MS was operated in positive EI mode at 70 eV with scanning over a mass acquisition range of 35–350 m/z. The standards solution of 62 commonly found compounds in wine were prepared at 13 scales dilutions in model wine solution (13% alcohol, titrate buffer, pH 3.2), and analyzed using the GC as wine samples to generate standard calibration curves for volatiles quantification. Wine volatiles were identified by comparing the mass spectra and retention indices with the NIST library in ChemStation and NIST Chemistry Webbook database and standard solutions obtained. All compounds were quantified based on GC peak ratio of individual compounds and internal standard, and the calibration curves generated from the standard solutions. The concentration of ethyl non-anoate (Quality control), blank SPME runs and blank internals standards were checked regularly.

### Statistical analysis

The grapevine canopy structure parameters, grape and wine biochemical test results and wine volatiles of different treatment groups were subjected to one-way analysis of variance (ANOVA) at *p* < 0.05 significance level (CoStat, version 6.4, CoHort software, Monterey, USA).

## Results and discussion

### Leaf area index analysis

**Figure 1:**
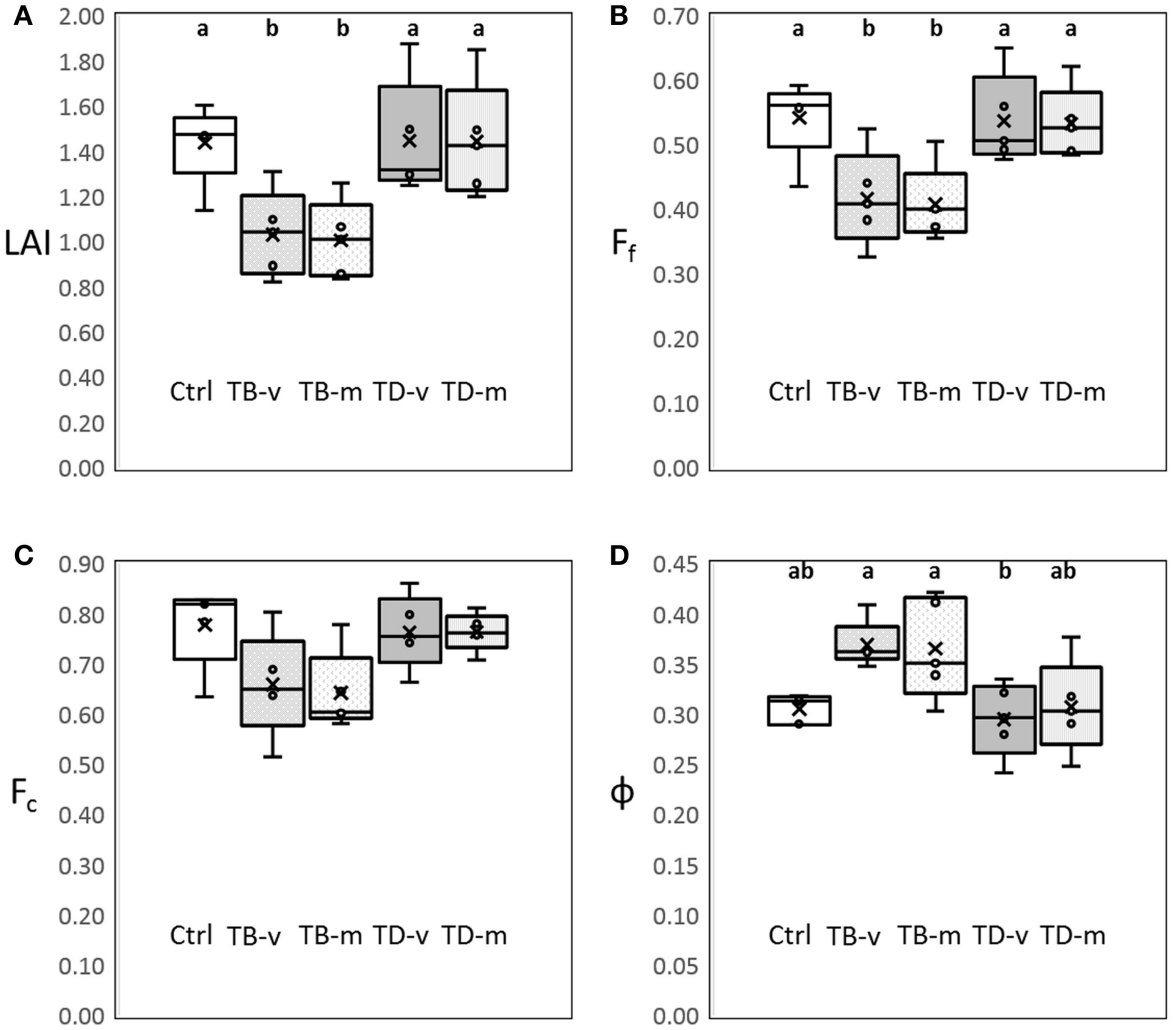
Influence of leaf trimming on canopy structure parameters measured at mid-ripeness in the 2015–2016 growing season: **(A)** leaf area index (LAI); **(B)** Foliage projective cover (Ff); **(C)** Crown cover (Fc); **(D)** Porosity (ϕ) (*p* < 0.05), no unites for each parameter. One-way ANOVA were conducted to compare different parameters at *p* < 0.05, and a, b were used to indicate statistically significant differences. Boxplot shows median value and standard deviation for each treatment groups. Control group (Ctrl), basal leaves removal at veraison (TB-v) at mid ripeness (TB-m), apical leaves removal at veraison (TD-v) at mid ripeness (TD-m).

The impacts of defoliation on the canopy structure of the grapevine was quantified using leaf area index (LAI), foliage projective cover (F_f_), crown cover (F_c_) and porosity (ϕ). Statistically significant lower LAI, F_f_ and higher ϕ were observed in basal defoliation groups (TB-v and TB-m), compared to the non-defoliated group (Ctrl; Figure 1). This study clearly quantifies the impacts of basal defoliation, with around 33% reduction in LAI, 27% reduction in F_f_ and 23% increase in ϕ (Figure 1). However, no significant differences were observed between Ctrl and apical defoliation groups (TA-v and TA-m) in the same parameters. No significant differences were observed amongst all experimental groups in F_c_ in the 2015–2016 growing season.

LAI and F_f_ explains the area of leave tissue and plant foliage per unit ground surface, respectively, while ϕ explains the light penetration rate through the canopy (Fuentes et al., 2008, 2014). Basal defoliation decreases the leaf area in the central part of the canopy and increase light penetration, and therefore explains the decrease in LAI and F_f_, and increase in ϕ. Minimal influences were observed in apical treatment groups, this was likely due to the VSP trellis system of the grapevine, where apical leaves mainly stay on top of the canopy. Therefore, apical defoliation has minimal impacts on grapevine canopy shading and canopy structure parameters. F_c_ represented area of the canopy per unit ground surface (Fuentes et al., 2008, 2014), and defoliation within the canopy does not influence the outer size of the canopy and therefore no significant differences were observed.

### Grape and wine composition analysis

Due to large variations amongst replicates, no statistically significant differences could be established among treatment groups in berry weigh and brix at the four sampling points (Table 1). However, there is a trend that apical defoliation tends to lead to slightly lower TSS (°Brix) at harvest in both seasons (2010–2011: Ctrl 22.00, TD-v 21.20; 2015–2016: Ctrl 23.2 ± 0.45, TD-v 22.9 ± 0.74, TD-m 22.5 ± 0.47). This trend can be confirmed by observing the alcohol strength (%v/v) of the finished wine (2010–2011: Ctrl 12.40, TD-v 11.72; 2015–2016: Ctrl 13.17 ± 0.69, TD-v 12.94 ± 0.43, TD-m 13.06 ± 0.17). Significant increases in the pH of harvested grape samples were observed in basal defoliation treatment at veraison compared to Ctrl (Table 1). Significantly decrease in TA was observed in all treatments in the 2015–2016 growing season and a similar trend was observed in the 2011–2012 growing season. The pH and TA was adjusted during winemaking process, and therefore pH and TA of the wine were not compared amongst experimental groups for finished wine. No significant changes were observed amongst experimental groups in the total anthocyanins and phenolics of grape berries at harvest in the 2015–2016 growing season, which is further confirmed in the wine profile where no differences were observed in wine color density, total phenolics, red pigments, color hue, and degree of red pigment coloration. However, reduction in grape total anthocyanins, wine color density and total red pigments were observed in TD-v treatment group compared to Ctrl in the 2010–2011 growing season.

**Table 1:** Summary of grape and wine chemical parameters.

	**Sampling time**	**2010–2011 vintage^a^**		**2015–2016 vintage^b^**				
		**Ctrl**	**TD-v^c^**	**Ctrl**	**TD-v**	**TB-v**	**TD-m**	**TB-m**
**GRAPE PARAMETERS**								
Berry weight (100 berries)	Veraison	161.6	148.0	81.0 ± 4.9	79.1 ± 8.0	86.0 ± 24.3	n/a^d^	n/a
	2 weeks Post Veraison	185.0	190.2	121.4 ± 13.4	129.28 ± 10.0	131.3 ± 12.7	126.6 ± 11.1	132.9 ± 6.7
	4 weeks Post Veraison	171.2	173.8	126.1 ± 9.5	128.8 ± 17.3	134.4 ± 16.1	123.8 ± 12.7	116.5 ± 20.5
	Harvest	159.8	164.4	107.4 ± 3.4	110.1 ± 9.2	119.5 ± 9.3	110.4 ± 8.2	113.8 ± 7.9
°Brix	Veraison	14.2	13.9	11.0 ± 0.3	11.4 ± 0.4	11.5 ± 0.5	n/a	n/a
	2 weeks Post Veraison	18.3	17.3	16.4 ± 1.4	15.7 ± 0.7	15.7 ± 0.5	15.5 ± 0.4	15.3 ± 0.3
	4 weeks Post Veraison	20.8	20.2	20.7 ± 0.5	20.7 ± 0.4	20.4 ± 1.0	20.6 ± 0.6	20.6 ± 0.8
	Harvest	22.0	21.2	23.2 ± 0.5	22.9 ± 0.7	23.2 ± 1.0	22.5 ± 0.5	22.6 ± 0.5
pH	Veraison	2.84	2.81	2.71 ± 0.04	2.71 ± 0.01	2.71 ± 0.06	n/a	n/a
	2 weeks Post Veraison	3.06	3.02	3.19 ± 0.08	3.17 ± 0.05	3.11 ± 0.09	3.12 ± 0.05	3.11 ± 0.03
	4 weeks Post Veraison	3.20	3.16	3.49 ± 0.02	3.5 ± 0.09	3.48 ± 0.1	3.51 ± 0.14	3.42 ± 0.04
	Harvest	3.36	3.39	3.79 ± 0.06b	3.94 ± 0.23ab	4.14 ± 0.22a	4.06 ± 0.1ab	4.01 ± 0.07ab
TA	Veraison	14.21	14.33	6.16 ± 0.35	6.38 ± 0.35	6.84 ± 0.35	n/a	n/a
	2 weeks Post Veraison	9.30	9.50	6.55 ± 0.85	6.42 ± 0.63	6.78 ± 0.56	7 ± 0.59	7.05 ± 0.08
	4 weeks Post Veraison	7.70	7.75	4.49 ± 0.08	4.48 ± 0.26	4.48 ± 0.29	4.43 ± 0.37	4.84 ± 0.37
	Harvest	7.25	6.60	3.84 ± 0.17a	3.06 ± 0.6b	2.6 ± 0.22b	2.59 ± 0.31b	2.85 ± 0.25b
Total anthocyanins (mg/g)	Harvest	1.68	1.28	0.7 ± 0.11	0.67 ± 0.06	0.62 ± 0.06	0.67 ± 0.08	0.64 ± 0.08
Total phenolics (a.u./g)	Harvest	n/a	n/a	0.35 ± 0.04	0.32 ± 0.02	0.32 ± 0.03	0.34 ± 0.02	0.31 ± 0.04
**WINE PARAMETERS**								
Total phenolics (a.u.)	Finished Wine	n/a	n/a	34.78 ± 5.91	35.27 ± 5.51	37.42 ± 5.49	34.78 ± 3.03	36.61 ± 3.63
Wine color density	Finished Wine	11.04	9.01	5.56 ± 0.8	5.61 ± 0.57	5.5 ± 0.22	5.47 ± 0.5	5.88 ± 0.6
Total red pigments (a.u.)	Finished Wine	16.78	13.06	11.24 ± 3.15	11.22 ± 2.26	12.33 ± 1.9	11.77 ± 1.36	12.31 ± 2.06
Wine color hue	Finished Wine	0.75	0.73	0.61 ± 0.04	0.62 ± 0.04	0.6 ± 0.04	0.58 ± 0.03	0.6 ± 0.03
Degree of red pigment coloration (%)	Finished Wine	37.67	40.00	31.86 ± 6.11	31.44 ± 3.51	28.21 ± 3.29	29.5 ± 2.56	30.11 ± 2.16
Alcohol (% v/v)	Finished Wine	12.40	11.72	13.27 ± 0.69	12.94 ± 0.43	13.28 ± 0.54	13.06 ± 0.17	13.28 ± 0.24

a *Replicates if Grape samples in each group in the 2010–2011 vintage were mixed in to one sample and analyzed for the biochemical composition*.

b *One-way ANOVA conduced for the samples in the 2015–2016 vintage at p < 0.05, a, b were used to indicate statistically significant differences*.

c *Control group (Ctrl), basal leaves removal at veraison (TB-v) at mid ripeness (TB-m), apical leaves removal at veraison (TD-v) at mid ripeness (TD-m)*.

d *n/a, not available*.

The impacts of defoliation on the composition of harvested grape has had varying reports and most of the research has focused on basal defoliation. Baiano et al. (2015) reported increased berry TSS and decreased berry TA, anthocyanins and phenolics in basal defoliation treatments at veraison and intermediate ripeness in Nero di Troia grapes. This is consistent to another study in Sauvignon Blanc and Riesling showing that basal leaf removal increase TSS and decrease TA and pH (Kozina et al., 2008). However, Di Profio et al. (2011) observed slightly increase of minimal changes in TSS, lower TA and higher pH in basal defoliation treatments at veraison in Merlot, Cabernet Franc and Cabernet Sauvignon. The same study also observed inconsistent results in berry anthocyanins and phenolics over three different vintages. A recent research reported that basal leaf removal at veraison did not significantly modify grape TSS, TA, anthocyanins and phenolics content in Merlot and Teran, but did slightly increase TSS and decrease TA in Plavac mali in one of the 2-year study (Osrečak et al., 2016). Similarly, another research in Merlot with basal leaf removal a pre-veraison showed no significantly differences in TSS, pH and TA, and increased anthocyanins in one season but decreased in another (King et al., 2012). Minimal changes in grape TSS, pH, and TA of basal defoliation treatment at pre-veraison and veraison were also observed in Pinot and Grenache (Tardáguila et al., 2008; Feng et al., 2015). Tardáguila et al. (2008) also reported minimal changes to total phenolics concent, while inconsistent results were observed over the 3 year trial in Pinot (Feng et al., 2015). Very few reports have studied the influences of apical leaf removal on grape content. Lanari et al. (2013) removed the apical part the canopy using mechanical leaf stripper at intermediate ripeness in Sangiovese and Montepulciano, and observed lower TSS in harvested grapes, which is consistent with the results presented here. The same study also observed lower anthocyanins and polyphenols in harvested Montepulciano, but not in Sangiovese. Another study in Sangiovese also observed lower berry TSS and wine alcohol level in treatment with defoliation in medium top part of the canopy, but minimal change in anthocyanins and phenolics, which is consistent to current results (Palliotti et al., 2013).

### HS-SPME-GC-MS analysis of wine volatiles

The volatile compounds in the wine samples of both vintage were analyzed using an HS-SPME-GC-MS method, which analyses 62 commonly found wine aroma compounds, and here only 17 volatile compounds were found of high concentrations in the sample wine (Table 2). The volatile compounds identified can classified as: fatty acid derived esters, other esters, alcohol derived acetate, and miscellaneous compounds. No statistically significant differences were observed amongst different treatment groups in the concentration of these volatile compounds, and large differences were observed between two vintages. Despite this, apical defoliaton resulted in much lower concentration of ethyl acetate, ethyl hexanoate, ethyl octanoate, ethyl decanoate, 3-methylbutyl acetate, and 1-hexanol in 2010–2011 vintage, but fewer differences were observed between Ctrl and TD-v in the 2015–2016 vintage. Higher concentration of diethyl butanedioate, ethyl 2-methylbutanoate, and ethyl 3-methylbutanoate were also observed in the apical defoliation group at veraison in the 2010–2011 vintage. However, in the 2015–2016 vintage the concentration of diethyl butanedioate in TD-v was lower than that of Ctrl, while ethyl 2-methylbutanoate and ethyl 3-methylbutanoate were not detected. All defoliation treatments had slightly higher average concentration of 1-heptanol and slightly lower average concentration of hexyl acetate, diethyl butanedioate, isobutanol, isopentanol, and ethyl 2-methylbutanoate, compared to non-defoliated group in the 2015–2016 vintage.

**Table 2:** Summary of HS-SPME-GC-MS results (*p* < 0.05).

**Peak number**	**LRI-Lit ^a^**	**LRI-Act^b^**	**Compound name**	**Odor^c^**	**2010–2011 vintage^d^^,^^e^**		**2015–2016 vintage^f^**				
					**Ctrl**	**TD-v^g^**	**Ctrl**	**TD-v**	**TB-v**	**TD-m**	**TB-m**
**ALCOHOL DERIVED ACETATE**											
1^h^	880	841	Ethyl acetate (μg/L)	Fruity, sweet, green	371	91	719 ± 358	636 ± 227	539 ± 204	719 ± 171	690 ± 193
6	1,126	1,111	3-Methylbutyl acetate (μg/L)	Fruity, sweet, banana	172	57	774 ± 117	778 ± 148	731 ± 195	810 ± 180	775 ± 96
9	1,272	1,270	Hexyl acetate (μg/L)	Fruity, green, apple	n/a	n/a	33 ± 19	27 ± 9	27 ± 10	29 ± 5	22 ± 5
**STRAIGHT-CHAIN ESTERS**											
2	1,034	1,016	Ethyl butanoate (μg/L)	Fruity, juicy, pineapple	215	212	593 ± 485	525 ± 236	385 ± 145	567 ± 365	638 ± 196
8	1,233	1,231	Ethyl hexanoate (mg/L)	sweet, Fruity, green	1,234	800	2.6 ± 0.4	2.9 ± 0.9	2.6 ± 0.3	2.8 ± 0.3	2.9 ± 0.2
14	1,439	1,435	Ethyl octanoate (mg/L)	waxy, Fruity, sweet	490	160	1.2 ± 0.3	1.1 ± 0.2	1.0 ± 0.1	1.1 ± 0.01	1.2 ± 0.2
17	1,638	1,638	Ethyl decanoate (μg/L)	Waxy, fruity, sweet	105	42	180 ± 41	182 ± 29	184 ± 23	171 ± 34	183 ± 28
**OTHER ESTERS**											
3	1,050	1,052	Ethyl 2-methylbutanoate (μg/L)	Apple, fruity, fresh	49	98	n/a	n/a	n/a	n/a	n/a
4	1,066	1,066	Ethyl 3-methylbutanoate (μg/L)	Sweet, apple, pineapple	87	149	n/a	n/a	n/a	n/a	n/a
10	1,339	1,345	Butyl lactate (μg/L)	Creamy, fruity, vanilla	n/a	n/a	34 ± 6	37 ± 12	37 ± 10	44 ± 13	32 ± 9
13	1,399	1,426	Ethyl 2-methyloctanoate (μg/L)	n/a	n/a	n/a	42 ± 8	38 ± 14	33 ± 4	36 ± 22	41 ± 17
18	1,680	1,677	Diethyl butanedioate (μg/L)	Fruity, apple, ylang	300	399	42 ± 14	37 ± 8	42 ± 4	34 ± 10	39 ± 8
**ALCOHOLS**											
5	1,094	1,085	Isobutanol (μg/L)	Whiskey, fusel, ethereal	71	73	96 ± 31	85 ± 22	80 ± 20	80 ± 7	78 ± 18
7	1,208	1,210	Isopentanol (mg/L)	Whiskey, malt, burnt	187	181	339 ± 15	347 ± 19	344 ± 18	345 ± 12	343 ± 24
11	1,355	1,358	1-Hexanol (mg/L)	Green, fruity, oily	4,358	2,901	5.3 ± 0.7	5.3 ± 1.3	4.5 ± 0.7	5.9 ± 1.1	5.5 ± 2.2
15	1,461	1,461	1-Heptanol (μg/L)	Green, fermented, nutty	n/a	n/a	158 ± 72	188 ± 131	187 ± 85	178 ± 178	222 ± 191
19	1,913	1,910	Phenylethyl Alcohol (mg/L)	Foral, rose, dried rose	22	21	15 ± 2	14 ± 3	15 ± 2	14 ± 2	12 ± 5

a *Linear retention index obtained from NIST chemistry webbook*.

b *Actual linear retention index calculated based on alkane standards*.

c *Odor of compounds obtained from Flavornet, The Good Scents Company and The Pherobase*.

d *Replicates if Grape samples in each group in the 2010–2011 vintage were mixed in to one sample and analyzed for the biochemical composition*.

e *Concentration of ethyl acetate, butyl lactate, diethyl butanedioate, isobutanol and ethyl 2-methyloctanoate were expressed as ug/L in 4-octanol correspondent, while ethyl hexanoate, ethyl octanoate, isopentanol, 1-hexanol and phenylethyl alcohol were expressed in mg/L*.

f *One-way ANOVA conduced for the samples in the 2015–2016 vintage at p < 0.05, a, b were used to indicate statistically significant differences*.

g *Internal standard 4-octanol (Peak 12) and quality control ethyl non-anoate (Peak 16) not shown in the table*.

h *Control group (Ctrl), basal leaves removal at veraison (TB-v) at mid ripeness (TB-m), apical leaves removal at veraison (TD-v) at mid ripeness (TD-m)*.

The influences of defoliation on wine volatiles profile are associated with five major factors, the first being different cultivars and clones having different responses to defoliation treatment. Significant reductions in the concentration of most wine volatile esters were observed in basal defoliated Sauvignon Blanc and Riesling, however Sauvignon Blanc is more sensitive to defoliation treatment and has much higher percentage of reduction (Kozina et al., 2008). Significant differences in wine volatile were reported amongst defoliation treatment groups of two different Sauvignon blanc clone (Šuklje et al., 2016). The timing of defoliation may have dramatic influence on grape composition, as shown by Šuklje et al. (2014) who showed that the influence of basal defoliation on Sauvignon Blanc was similar as that of Kozina et al. (2008) (treatment at veraison), but when performed at an earlier berry development stage (treatment at pepper core size), observed higher concentration of volatile esters in Sauvignon Blanc. Similarly, another study in Tempranillo reported much higher total acetates and lower wine alcohols in defoliation group at pre-bloom compared to fruit-set (Vilanova et al., 2012). Defoliation by mechanical or manual methods are another factor may affect volatiles profile of the resultant wine. Dramatic reduction of wine volatile esters were only observed in manual defoliation treatment, but not in mechanical treatment groups in Tempranillo (Vilanova et al., 2012). Further harvest time can also alter the result of defoliation. Significantly higher concentrations of wine esters were observed in the defoliation treatment group at first harvest, but no significant differences when a second harvest was conducted 12 days later (Verzera et al., 2016). Finally, the location of the defoliation as given in the current study is another cause of variation in wine volatiles. This study is the first to study the effect of apical defoliation, but together with two studies shows that apical leaf removal modifies grape berry composition differently compared to basal leaf removal and therefore influences wine volatiles differently (Lanari et al., 2013; Palliotti et al., 2013).

The influences of apical defoliation on grape volatiles such as terpenes, have yet to be elucidated. This is especially true when terpenoid compounds, such as rotundone, are associated with wine quality (Herderich et al., 2013, 2015). Since terpenes may be produced differently within individual bunches, individual grapevines, and individual vineyard blocks (Zhang et al., 2013, 2015a, 2016c), it is essential to consider the location and aspects of the vineyard when conducting defoliation treatments. Additionally, canopy defoliation is associated with other factors, such as solar exposure, which is further associated with bunch zone microclimate (Zhang et al., 2015b). Thus, these factors have the potential to influence the defoliation outcome and therefore should be considered separately as suggested previously (Spayd et al., 2002). More importantly, terpenoids in grape berries are actively produced in different phenological stages (Zhang et al., 2016a,b), and therefore apical defoliation at different phenological stages should also be investigated.

## Conclusions

Defoliation is a commonly used technique to manipulate grape and wine composition, and the present study compared both basal defoliation and the less studied apical defoliation and its influences on grape and wine composition. Compared to basal defoliation, apical defoliation has less influence on the grapevine canopy and therefore less potential effect on the bunch zone microclimate. Apical defoliation near veraison resulted in slightly increased pH and decreased TA in harvested grape berries, and could lower the alcoholic strength in the resultant wine. Reduction of wine anthocyanins and color profile in apical treatment groups were observed in one season, but not the other. Minimal changes were observed in major wine volatile compounds amongst non-defoliation and defoliation treatments. These results demonstrate that apical defoliation is a novel and effective way to moderate wine alcohol with minimal influenced on wine aromatic properties, and therefore may be a technique to mitigate the influences of global warming on increasing wine alcohol level. Further research on apical defoliation is needed to study the influence on other important aromatic compounds. The timing and location of apical defoliation and its interaction with other viticulture and climate factors need to be investigated to determine the optimal apical defoliation technique suitable for commercial vineyards.

## Author contributions

PZ and KH conceived and designed the study, analyzed the data, and wrote the paper. SF and DL contributed analytical methods and finalized the data. XW, SN, and PZ performed the experiments. PZ, KH, SF, SN, and XW participated in the design and critical revision. All authors read and approved the final manuscript.

### Conflict of interest statement

The authors declare that the research was conducted in the absence of any commercial or financial relationships that could be construed as a potential conflict of interest.
